# Impact of metric and sample size on determining malaria hotspot boundaries

**DOI:** 10.1038/srep45849

**Published:** 2017-04-12

**Authors:** Gillian H. Stresman, Emanuele Giorgi, Amrish Baidjoe, Phil Knight, Wycliffe Odongo, Chrispin Owaga, Shehu Shagari, Euniah Makori, Jennifer Stevenson, Chris Drakeley, Jonathan Cox, Teun Bousema, Peter J. Diggle

**Affiliations:** 1 Department of Infectious and Tropical Diseases, London School of Hygiene & Tropical Medicine, London, United Kingdom; 2 Faculty of Health and Medicine, Furness College, Lancaster University, Lancaster, United Kingdom; 3 Radboud University Medical Center, Nijmegen, the Netherlands; 4 Department of Ecology and Geography, University of Bath, Bath, United Kingdom; 5 Kenya Medical Research Institute, Centre for Global Health Research, Kisumu, Kenya; 6 Malaria Centre, Johns Hopkins Bloomberg School of Public Health, Baltimore, United States; 7 Institute of Infection and Global Health, University of Liverpool, Liverpool, United Kingdom

## Abstract

The spatial heterogeneity of malaria suggests that interventions may be targeted for maximum impact. It is unclear to what extent different metrics lead to consistent delineation of hotspot boundaries. Using data from a large community-based malaria survey in the western Kenyan highlands, we assessed the agreement between a model-based geostatistical (MBG) approach to detect hotspots using *Plasmodium falciparum* parasite prevalence and serological evidence for exposure. Malaria transmission was widespread and highly heterogeneous with one third of the total population living in hotspots regardless of metric tested. Moderate agreement (Kappa = 0.424) was observed between hotspots defined based on parasite prevalence by polymerase chain reaction (PCR)- and the prevalence of antibodies to two *P. falciparum* antigens (MSP-1, AMA-1). While numerous biologically plausible hotspots were identified, their detection strongly relied on the proportion of the population sampled. When only 3% of the population was sampled, no PCR derived hotspots were reliably detected and at least 21% of the population was needed for reliable results. Similar results were observed for hotspots of seroprevalence. Hotspot boundaries are driven by the malaria diagnostic and sample size used to inform the model. These findings warn against the simplistic use of spatial analysis on available data to target malaria interventions in areas where hotspot boundaries are uncertain.

Malaria is an important cause of global morbidity and mortality with an estimated 3.4 billion people at risk^1^. The past decade has seen a large reduction in the malaria burden in some areas with an estimated 47% global reduction in mortality compared to 2000^2^. As national policies shift from control towards elimination new approaches are needed to supplement existing tools^3^,^4^. Research and programmatic activities are increasingly acknowledging the heterogeneous nature of malaria transmission at the community level.

Identifying ‘hotspots’ and targeting malaria control interventions at these, could lead to a more sustainable reduction in malaria burden^5^,^6^. Hotspots are typically defined in both public health and ecology as areas where estimates exceed those from other areas and may fuel transmission to the surrounding areas^5^,^7^,^8^. Malaria transmission is difficult to measure directly and several metrics are typically used to estimate malaria burden as a proxy for transmission^9^. However, different malaria metrics measure different facets of the transmission cycle and may lead to different conclusions on the existence, size or location of hotspots. For example, in coastal Kenya hotspots based on clinical incidence were geographically distinct and showed different temporal dynamics compared to hotspots based on the prevalence of asymptomatic infections^10^,^11^.

The detection of malaria hotspots has become increasingly prominent in the malaria literature^12^,^13^,^14^,^15^,^16^,^17^. Model-based geostatistics (MBG) are increasingly being used to identify heterogeneity in malaria transmission and can predict areas of increased disease prevalence. MBG has been effectively applied in other disease systems that exhibit both large and small-scale variation in transmission^18^,^19^. In the context of malaria, MBG has mainly been applied at the national or provincial scales, although it has yet to be widely applied for local level spatial analysis^13^,^20^,^21^. It allows incorporating environmental drivers of disease transmission and information on the intensity of sampling to obtain smoothed values of disease indicators to determine spatial patterns in disease occurrence. Determining the hotspot boundaries is of great public health importance if hotspot-targeted interventions are considered. Uncertainties about hotspot boundaries would complicate and potentially reduce the impact of hotspot-targeted interventions by potentially missing populations that are particularly relevant for onward transmission or misallocating resources^22^.

Using data collected in a large cross-sectional malaria survey carried out in the western Kenyan highlands, the aims of this study were to compare the agreement between spatial analysis based on the prevalence of molecularly detected malaria infections and serological evidence for malaria exposure and illustrate the impact of sample size on the delineation of hotspots of malaria. The results generated are not intended to provide a gold standard for hotspot detection, but to illustrate the realities of translating theoretical concepts of disease heterogeneity into actionable public health strategies.

## Methods

### Data sources

#### Epidemiological

Epidemiological data were obtained from a community cross-sectional malaria survey conducted in July 2011 in a 100 km^2^ rural area in the western Kenyan highlands (0°28′S, 34°51′E)^23^. The site is characterized by low but heterogeneous malaria transmission, with *Plasmodium falciparum* being the predominant species^24^. Factors determining local malaria transmission patterns were recently described^25^.

All structures in the study area were digitized using high-resolution satellite imagery (Quickbird, DigitalGlobe Services Inc, USA) and were used as a proxy for the total population size and distribution^22^,^23^. Briefly, 17,503 individuals residing in 3,213 randomly selected households (i.e. clusters of structures forming a family unit), or approximately 30% of the total population, were surveyed, with each participant providing blood spot samples on filter paper. The unit of analysis was the household with the proportion of household residents that were positive for malaria the main outcome. A random selection of 79% of the collected samples were assayed by PCR to detect the presence of a current malaria infection, corresponding to an estimated 24% of the total population^26^,^27^; all samples were tested for anti-malarial antibody response to AMA1 and/or MSP1_19_ measured by enzyme linked immunosorbent assay to provide a measure of malaria exposure^28^,^29^. Seropositivity to each antigen was assessed using a mixture model and consisted of those individuals with optical density values greater than the mean plus three standard deviations of the distribution of those assumed negative^30^. An individual was considered to be seropositive if they were positive to either or both of the antigens tested. Ethical approval for collecting the epidemiological data was granted by the London School of Hygiene & Tropical Medicine (LSHTM-5721) and the Kenya Medical Research Institute (SSC-1802). All methods were performed in accordance with good research practices and written informed consent was obtained from all participants.

#### Environmental

Elevation for each household was derived from the ASTER [v 2.0, NASA USA] global digital elevation model (DEM). The normalized-difference vegetation index (NDVI) was calculated for the study area using the Quickbird imagery. Mean, minimum, and maximum NDVI values from a single time-point were calculated for a 500 m circular buffer around each household. Multispectral image segmentation (MIS) of the Quickbird imagery was conducted with eCognition (v 4.0, Trimble Geospatial Imaging, Germany) software and the proportion of tree cover within the 500 m buffer was determined. Fishponds were identified using a refined MIS procedure capable of detecting smaller features and manually verified against the satellite imagery. The distance from each household to the nearest fishpond was calculated in ArcGIS (ESRI, USA).

Topographic wetness index (TWI) was calculated using the DEM as previously described^31^. The maximum and mean TWI values for the 500 m surrounding each household were calculated. Finally, the locations of all streams in the area were determined by first locating the likely location of streams using the topographic data and then manually digitizing the more precise stream path using the satellite imagery. The distances of each household to all stream orders were calculated^32^.

### Determining hotspots of P. falciparum infection and exposure by MBG

MBG was used to model the spatial variation in malaria parasite or antibody prevalence^18^,^19^. Two models were generated using the PrevMap package^33^: malaria infection was assessed using PCR positivity and exposure to malaria was assessed using seropositivity estimates for each household (see Supplementary file 1)^34^. Surfaces of predicted prevalence for both outcomes were generated. To guide MBG, thresholds of risks were used that resulted in 20% of the population being included in the hotspot based on the theoretical 80–20 assumption where 20% of the population constitutes 80% of the exposure and transmission events^35^. We acknowledge that this threshold selection is likely to be site specific and the hotspot sizes will vary based on the threshold selected: a high threshold would result in only those areas with the highest transmission being identified as a hotspot and a more granular map whereas a less stringent threshold would mean that hotspots would be more ubiquitous.

Next, the probability that any given area exceeded the threshold that encompassed 20% of the population was determined. Areas with greater than 80% probability of exceeding the threshold were considered hotspots. In an ideal scenario, the model will produce a probability surface that is polarized into areas with 100 or 0% probability of exceeding a specified threshold. The 80% probability threshold was selected to capture those areas that are almost certainly in a hotspot as well as including those most likely to be in a hotspot, a decision made to favour a higher sensitivity rather than specificity. To gauge the sensitivity of the exceedance threshold in determining hotspots, we also identified areas that had greater than 50% probability of exceeding the threshold (i.e. any likelihood of being a hotspot). This process was repeated for both outcomes to generate separate surfaces for hotspots of current infection and exposure to malaria and there were no constraints placed on hotspot size or shape. The households consistently identified, or agreement, between hotspots of infection and exposure was assessed using Cohen’s Kappa coefficient. All analyses were conducted in R v.3.0.2 (R-Project, USA).

### Sample size

The impact of sample size on the ability of the model to predict hotspots and therefore confidence in delineation of hotspot boundaries, was assessed by the change in metrics of model predictive performance: the integrated mean square error (IMSE) for the predicted surface and the discrimination index (DI) for the exceedance probabilities^18^. To estimate the level of performance that would have been achieved had the entire (100%) population been sampled, we imputed a complete population data set using the complete set of digitized households and the predicted malaria risk surfaces to estimate household level prevalence^23^. Next, we selected a random sub-set of the imputed data for each of the sampling fractions 10–90% and re-fitted the geostatistical models to each sub-set. The corresponding IMSE and DI values were calculated and plotted as functions of the sampling fraction.

To determine the impact of sample size on hotspot boundaries, the geostatistical model was then re-fitted to random subsets of the collected data, with sampling fractions between 10–90%. The resulting surfaces were imported into ArcGIS, hotspot boundaries determined, and individual households were assigned as hotspot or non-hotspot accordingly. The sensitivity and specificity of the structures correctly identified, using the complete sample as the reference, were calculated and compared using the area under the receiver operator curve (AUROC)^36^. A meaningful change was considered to be those with non-overlapping confidence intervals for the AUROC.

## Results

### MBG models

The results of the geostatistical model are consistent with previous studies and suggests a positive association between parasite prevalence and maximum and mean NDVI and a negative association with mean elevation, distance from fishponds and the proportion of tree cover (see Supplementary file 2 for model validation)^22^,^25^. The optimum model fit for seroprevalence also indicated a negative association with mean elevation, distance from fishponds and tree cover. In addition, maximum TWI, minimum NDVI and distance to 2^nd^ and 3^rd^ order streams had negative associations with seroprevalence, while mean TWI had a positive association (Table 1). The percentage of variability explained by the covariates was 3% for parasite prevalence and 18% for seroprevalence. The spatial stochastic process, which accounts for both local and global spatial trends, accounted for 53.4% and 49.5% of the unexplained variability according to PCR and seropositivity, respectively.

### Comparing metrics: Molecular vs. Seroprevalence

**Figure 1:**
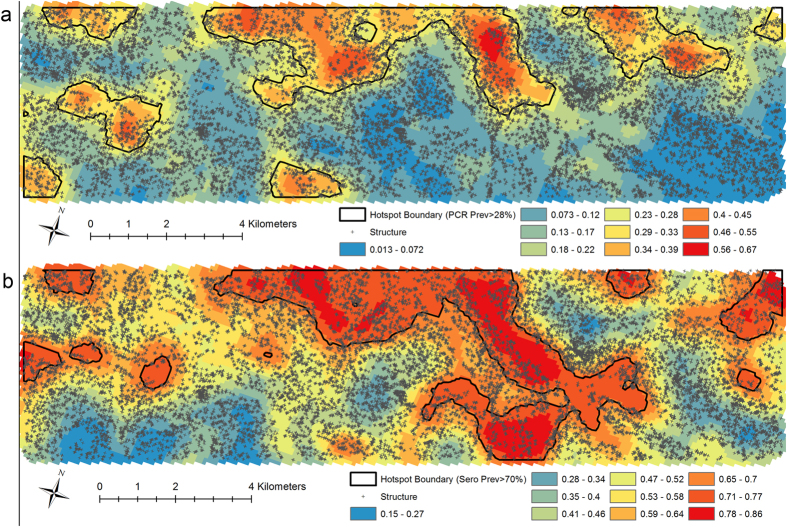
Predicted malaria prevalence using model based geostatistics. Results of the modeled predicted prevalence of (**a**) current malaria infection with overlaid hotspot boundaries showing the area that has a predicted PCR prevalence greater than 28% and (**b**) malaria exposure as measured by seroprevalence with overlaid hotspot boundaries showing the area that has a predicted seroprevalence greater than 70%. Maps were generated using the PrevMap package in the R statistical software (V3.0.2 R-Project USA).

**Figure 2:**
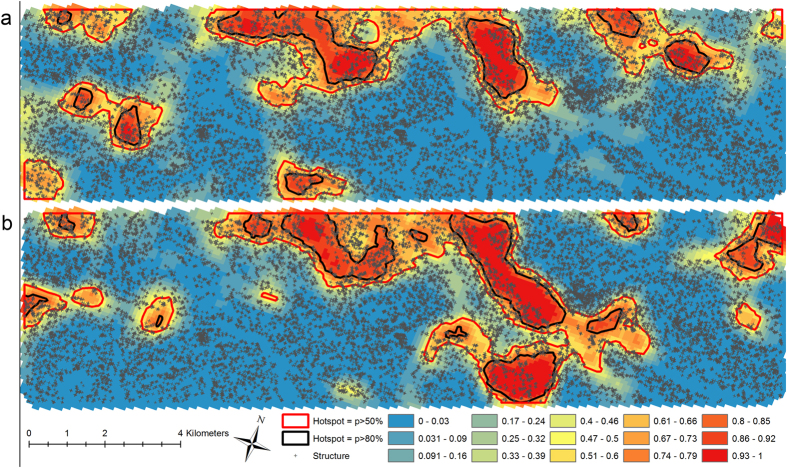
Probability contour maps of exceedance surfaces for malaria prevalence. Contour maps of the study area indicating the probability that the prevalence of malaria (**a**) infection by PCR and (**b**) exposure by seroprevalence exceeds 28% and 70%, respectively with the corresponding hotspot boundaries using both 50% and 80% thresholds. Maps were generated using the PrevMap package in the R statistical software (V3.0.2 R-Project USA).

Areas with a predicted PCR prevalence (Fig. 1a) greater than 28% and predicted seroprevalence (Fig. 1b) greater than 70% encompassed 20% of the total population. These thresholds were subsequently used to determine hotspots of infection and exposure, respectively, for the MBG approach. The probability of exceeding the defined thresholds was mapped for both current infection prevalence (Fig. 2a) and previous exposure, seropositivity (Fig. 2b). The agreement between households identified as part of hotspots derived using parasite and sero-prevalence with a probability >80% of exceeding the threshold was moderate (Kappa = 0.424). Using hotspot boundaries corresponding to areas with >50% probability of exceeding the threshold resulted in only modest improvement in agreement (Kappa = 0.478).

### Impact of sample size

**Figure 3:**
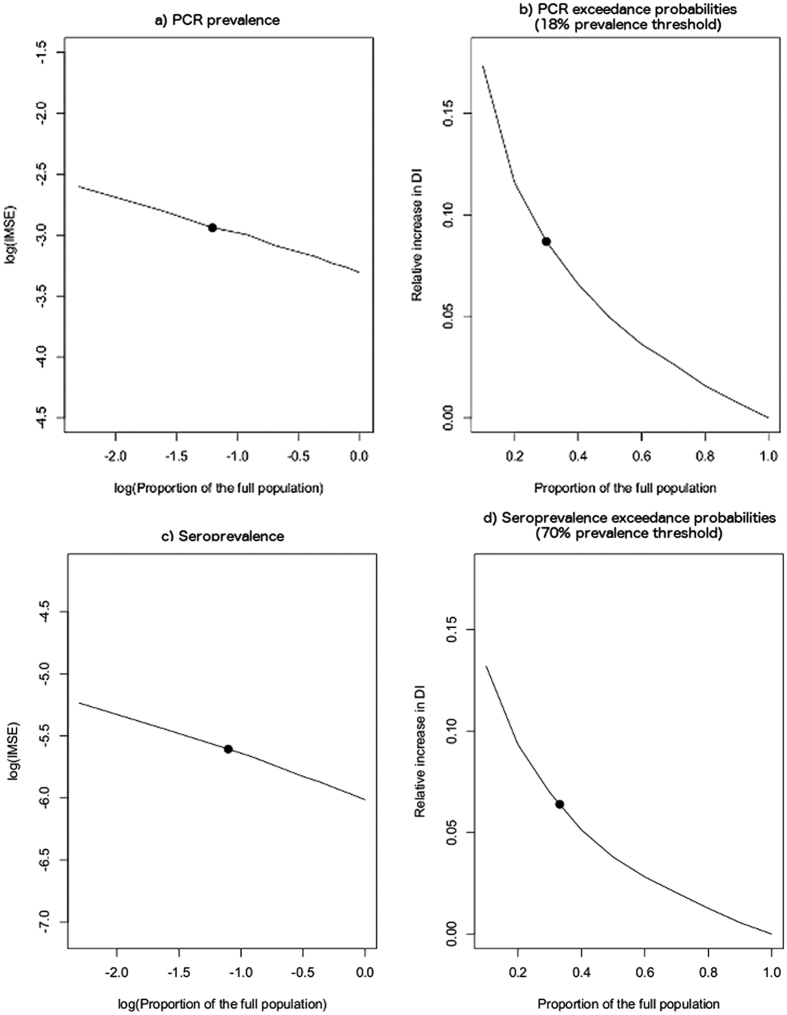
Impact of sample size on geostatistical model efficiency. The impact of reduced sample size on model efficiency on the log-scale for both the predicted and probability surfaces for both PCR (**a**,**b**) and seroprevalence (**c**,**d**), respectively (solid line) with the circle representing the sample size achieved during the community survey.

As expected, IMSE decreased proportionally to the inverse of the sample size (Fig. 3). Based on this analysis, the models using the survey data to generate predictive surfaces were generated with an estimated baseline error of a 40% relative increase in IMSE.

The parasite prevalence model showed a change in the number of structures correctly identified when sample size was reduced to 70% of the sampled population, or 21% of the total population (Table 2). A second significant difference in the consistency of hotspot delineation was observed with 30% of the sampled population, or 9% of the total population (Supplementary movie 1). The geostatistical model for parasite prevalence was unable to reliably detect hotspots with less than 10% of the sampled, or 3% of the total population. The impact of sample size on the models for seroprevalence showed similar trends in terms of the proportion of the population required to consistently define hotspots. (Supplementary movie 2).

## Discussion

We present a MBG analysis to define hotspots in an area with known highly heterogeneous transmission in the western Kenyan highlands. Different metrics and sample sizes resulted in variation in the households identified as being located in a hotspot. These results illustrate the uncertainties in determining precise boundaries at the local level that may be relevant for targeted control intervention.

This study utilized two metrics for defining malaria heterogeneity, the prevalence of current infections and the prevalence of serological markers that are indicative of previous exposure. These metrics measure different but analogous facets of malaria transmission and at an individual level are strongly associated^22^,^25^. In the current study and other studies determining spatial patterns in malaria transmission, the localization of hotspots based on the two metrics showed considerable overlap but imperfect agreement. Recent or transient malaria hotspots may be missed by serological markers of exposure if antibodies are only detectable following repeated exposure to malaria antigens^10^,^37^. However, serologically defined hotspots would be a more stable representation of areas of consistently or historically high risk but may not reflect areas with recent infections^38^,^39^. Recent advances in identifying serological markers of recent exposure may improve the agreement between serology and current parasite prevalence^40^ although it remains to be established whether these are as informative as the currently used antigens that show high sensitivity in low endemic settings^41^.

For both metrics, only a small percentage of the geographical variability that we observed in our study population was explained by environmental covariates: the spatial residuals estimated by the geostatistical model formed the main component in identifying hotspots. Obtaining a better understanding of the spatial processes driving transmission and identifying covariates accordingly would lead to more precise delineation of MBG-defined hotspots. The specific ecological processing driving transmission will be setting dependent and the necessity of delineating this spatial dynamic will depend on how well these approaches are able to identify hotspots that are meaningful for control and elimination strategies.

As expected, variations in sample size also resulted in significant changes in the boundaries of MBG-defined hotspots. In our setting, intensive sampling of the total population was conducted for accurate hotspot delineation. If less than 20.9% of the total population was sampled (70% of all samples available for our survey), a considerable loss in accuracy was experienced. An average of 37.6% (seroprevalence) and 51.4% (PCR-prevalence) of the structures in hotspots were misclassified as not belonging to hotspots while the complete sample set identified them as hotspots. This suggests that one third to half of structures may be missed by interventions designed to target hotspots or unnecessarily targeted. The sample size thresholds identified here are likely not generalizable to other settings. The purpose of this element of the current study was to illustrate that where the hotspots are drawn will be impacted by the sample size used to inform the analysis. The current findings warn against conducting opportunistic analysis on available geocoded data^7^,^42^ if these data were only available for a small fraction of the population.

Although this comparison of metrics cannot determine which is better able to accurately identify and define true hotspots of infection in the community, these results indicate that the approach and assumptions used will affect the resulting map. The MBG approach is generally used to fit a spatial residual risk surface. It allows for a greater understanding of the nature of malaria hotspots by letting the overall risk surface depend on both measured and unmeasured risk factors^18^. However, the underlying inferential philosophy of MBG is that it is concerned not with how likely it is that a location has an above-average prevalence, but with how likely it is that a given location has a prevalence sufficiently high to be of practical concern in a specific setting. Therefore, the thresholds defined for both the prevalence of concern as well as the probability cut-off that an area has reached or exceeded that must be identified and affects the resulting map. This feature provides useful flexibility so that this approach can easily be tailored to different settings, but also makes it difficult to identify precise hotspots if such policy thresholds do not exist^19^,^43^.

## Conclusions

This research has highlighted several gaps in our ability to reliably detect hotspots of malaria. The metric and sample size used has important consequences for hotspot boundaries in this setting. The operationally most attractive approach of sampling a small fraction of the population and use the most scalable and economically attractive malaria metric, in our study serology, has limitations in terms of the precision with which hotspot boundaries can be identified. It is important to note that the study setting was characterized by low, heterogeneous but widespread malaria transmission. There may be settings where malaria is more focal, where hotspots are more readily detectable and more consistent between metrics. For our study setting with widespread heterogeneous malaria transmission, we conclude that there are too many uncertainties surrounding hotspot location, stability and boundaries to allow evidence-based targeting of malaria hotspots with the aim of reducing community-wide malaria transmission.

## Additional Information

**How to cite this article:** Stresman, G. H. *et al*. Impact of metric and sample size on determining malaria hotspot boundaries. *Sci. Rep.* **7**, 45849; doi: 10.1038/srep45849 (2017).

**Publisher's note:** Springer Nature remains neutral with regard to jurisdictional claims in published maps and institutional affiliations.

## Supplementary Material

Supplementary Movie 1

Supplementary Movie 2

Supplementary Files

## Figures and Tables

**Table 1:** Final adjusted mixed effects logistic regression models for both outcomes.

PCR Prevalence				Seroprevalence			
	Estimate	Std. error	p.value		Estimate	Std. error	p.value
Intercept	5.430	3.272	0.097	Intercept	7.972	2.165	0.0002
Mean Elevation	−0.007	0.002	<0.0001	Mean Elevation	−0.005	0.001	<0.0001
Maximum NDVI	1.532	1.030	0.137	Max TWI	−0.011	0.011	0.297
Mean NDVI	5.132	2.934	0.080	Mean TWI	0.230	0.104	0.028
Distance from Fish Pond	−0.001	0.000	0.000	Minimum NDVI	−0.227	0.229	0.320
Tree Cover	−3.094	1.473	0.036	Distance from Fish Ponds	−0.0005	0.0001	<0.0001
				Distance 3^rd^ Order Stream	−0.0001	0.000	0.039
				Distance 2^nd^ Order Stream	−0.0002	0.0001	<0.0001
				Tree Cover	−2.921	0.8194	0.0004

**Table 2:** Results of the impact of sample size on the ability to consistently detect the same structures as being located inside hotspots of malaria infection (PCR prevalence) and exposure (seroprevalence). AUROC = Area Under the Receiver Operator Curve.

% of Sample	PCR Prevalence				Seroprevalence			
	% of Total Population	AUROC	Std. Error	95% CI	% of Total Population	AUROC	Std. Error	95% CI
100	29.9	1.0	—	—	33.2	1.0	—	—
90	26.9	0.923	0.0048	0.914–0.933	29.9	0.926	0.0039	0.918–0.934
80	23.9	0.896	0.0054	0.885–0.906	26.6	0.913	0.0042	0.905–0.921
70	20.9	0.847	0.0061	0.835–0.859	23.4	0.859	0.0050	0.849–0.869
60	17.9	0.812	0.0065	0.799–0.824	19.9	0.855	0.0050	0.845–0.865
50	14.9	0.819	0.0064	0.807–0.832	16.6	0.866	0.0049	0.856–0.875
40	12.0	0.834	0.0062	0.821–0.846	13.3	0.773	0.0056	0.761–0.784
30	9.0	0.739	0.0067	0.726–0.752	10.0	0.804	0.0054	0.793–0.815
20	6.0	0.693	0.0066	0.680–0.706	6.6	0.706	0.0056	0.695–0.717
10	3.0	—	—	—	3.3	0.744	0.0057	0.733–0.755
