# Supplementary material for: Impact of metric and sample size on determining malaria hotspot boundaries
Source: Sci Rep. 2017 Apr 12;7:45849. doi: 10.1038/srep45849 (PMC5388846; doi:10.1038/srep45849)
Supplement: Supplementary Files [file srep45849-s3.doc]

**Title**: Impact of metric and sample size on determining malaria hotspot boundaries

**Authors**: Gillian H Stresman*^❖1^, Emanuele Giorgi*^2^, Amrish Baidjoe^3^, Phil Knight^4^, Wycliffe Odongo^5^, Chrispin Owaga^5^, Shehu Shagari^5^, Euniah Makori^5^, Jennifer Stevenson^1,5,6^, Chris Drakeley^1^, Jonathan Cox^1^, Teun Bousema^§ 1,3^ , Peter J Diggle^§ 2,7^

*Authors Contributed Equally

^§^ Authors Contributed Equally

^❖^Correspondence to Gillian.Stresman@lshtm.ac.uk

**Affiliations:**

1 – Department of Infectious and Tropical Diseases, London School of Hygiene & Tropical Medicine, London United Kingdom

2 – Faculty of Health and Medicine, Furness College, Lancaster University, Lancaster United Kingdom

3 - Radboud University Medical Center, Nijmegen, the Netherlands

4 – Department of Ecology and Geography, University of Bath, Bath United Kingdom

5 – Kenya Medical Research Institute, Centre for Global Health Research, Kisumu, Kenya

6 – Malaria Centre, Johns Hopkins Bloomberg School of Public Health, Baltimore, United States

7 – Institute of Infection and Global Health, University of Liverpool, Liverpool, United Kingdom

**Supplementary File 1**

This document outlines the underlying statistical methodology of the “Methods” section in the paper. In the remainder of the document we will refer both to PCR and seroprevalence as “test”. In Section 1 we describe the geostatistical models that were fitted to the PCR and seroprevalence data. In Section 2, we give some details on the metrics used for the sample size calculations.

**1 Geostatistical analysis**

**1.1 Model**

Let *Y_i_* denote the number of positive counts of the test in the i-th household, each associated with sampling locations *x_i_*, for *i*=1, . . . , *n*. Conditionally on the realization of the random effect *T(x_i_),* the response variable *Y_i_* follow a Binomial distribution with expected value *E[Y_i_] = n_i_p_i_* where *n_i_* is the number of household members and *p_i_* is the probability of having a positive test. We use the canonical logit link function defined as

(1)

where *d(x_i_)* is a vector of spatial covariates with associated vector of regression coefficients *β*; *S(x_i_)* is a stationary isotropic zero-mean Gaussian process with variance *σ^2^* and correlation function *ρ(u) =* exp*(-u/Φ)* with *u* being the distance between two households and scale parameter *Φ* > 0; and *Z_i_* are mutually independent Gaussian variables that are used to account for non-spatial variation within housholds.

The set of spatial covariates *d(x_i_)* used in the model were selected using ordinary logistic regression and that were significant at 5% confidence level; in table 1, these are reported indicating their inclusion in the PCR and seroprevalence models.

|  | Term | PCR | Seroprevalence |
| --- | --- | --- | --- |
| 1 | Intercept | Yes | Yes |
| 2 | Mean elevation | Yes | Yes |
| 3 | Maximum NDVI | Yes | No |
| 4 | Mean NDVI | Yes | No |
| 5 | Distance from closest fish pond | Yes | Yes |
| 6 | Tree cover | Yes | Yes |
| 7 | Maximum TWI | No | Yes |
| 8 | Mean TWI | No | Yes |
| 9 | Distance from the 3^rd^ order stream | No | Yes |
| 10 | Distance from the 2^nd^ order stream | No | Yes |
| Table 1: Identified spatial covariates using an ordinary logistic regression; third and fourth columns indicate their presence in the models for PCR and seroprevalence, respectively | | | |

**1.2 Parameter estimation**

We use the Monte Carlo maximum likelihood (MCML) method (Geyer & Thompson, 1992; Geyer, 1994, 1996, 1999) for estimation of the model parameters. This procedure uses conditional simulations of the random effect *T* given the data *Y* to obtain a computationally efficient approximation to the intractable likelihood function. More details on the analytical derivation of such an approximation can be found in Christensen (2004) and Giorgi et al. (2015).

1.3 Prediction

Now, consider the prediction of *T^*^*=*(T*(*x_n+1_*)*,* . . . , *T*(*x_n+q_*))*^Τ^* at *q* additional prediction locations forming a regular grid at spacing 100 m over the entire surveyed area. All relevant explanatory variables, listed in Table 1, were also available at the prediction locations. We do not include the mutually independent random variables *Z_i_* in 1 as part of our target for prediction, since, in our case, these are interpreted as non-spatial variation within households.

Using a Monte Carlo Markov chain algorithm proposed by Christensen et al. (2006), we obtain 10^4^ samples from the distribution of *T^*^* given *Y* by simulating 110000 samples and retaining every 10^th^ sample after a burn-in of 10^4^ simulations. Let *t_(1)_*(*x_n+i_*)*, . . . , t_(10_^4^)*(*x_n+i_*), denote the simulated samples for the *i*-th grid locations *x_n+i_*. Predicted prevalences are obtained by transforming the sampled values *t_(j)_*(*x_n+i_*) to *p_(j)_*(*x_n+i_*) = exp{*t_(j)_(x_n-i_*)}/(1+exp{*t_(j)_*(*x_n+i_*)}) for *i*=1, . . . , *q* and *j*=1, . . . , 10^4^. We then summarize the resulting set of predicted prevalence surfaces with the following indices.

- Point-wise mean are obtained as
- Exceedance probabilities are obtained as , for *i*=1, . . . , *q* where *c* is a pre-defined prevalence threshold (*c* = 0.28 for PCR and *c* = 0.70 for seroprevalence) and *I_(c,1)_*{*p*} is an indicator function that takes value 1 if *c* < *p* < 1 and 0 otherwise.

**2 Computational details of the sample size calculations**

Let *A* denote the surveyed region of interest in R^2^, *μ*(*x*) = *d*(*x*)*^T^β* the fixed effect part of the linear predictor in (1) and define *Ŝp*(*x*) to be the kriging predictor of *S*(*x*) obtained by a sample corresponding to (100 x *p*)% of the total population. The integrated mean-square error (IMSE) and the discrimination index (DI) are the defined as follows (Fanshawe & Diggle, 2013)

(2)

(3)

where *E*{•} is the expected value with respect to the distribution of *S*(*x*) and *l* = log(*c*/(1 - *c*)), with *c* given prevalence threshold as specified in Section 1.3. The IMSE index in (2) quantifies the overall mean-square error in *A* of the odds ratio spatial predictor. In (3), DI measures how well the design of a given sample size discriminates hotspots; under ideal circumstances all the predictive probabilities would be either 0 or 1.

In order to compute the intractable integrals in (2) and (3), we impose the spatially continuous process *S*(*x*) to be piecewise constant over a regular grid (*x_1_*, . . . , *x_N_*) in *A* at spacing 220 m. For a given proportion *p* of the total population, we then compute the IMSE and DI metrics using the following Monte Carlo procedure.

1. Select randomly a set of locations corresponding to (100 x *p*)% of the total digitized structures aggregated to the household level, with the distance between any two sampled locations no less than 20 m in order to guarantee a good spatial coverage in *A*.
2. Simulate 10^4^ surfaces of *S*(*x*) over the regular grid in *A*, setting the covariance parameters equal to the respective MCML estimates (see Section 1.2). Let now be the *i*-th simulated surface.
3. For each of the randomly chosen locations obtained from 1, select *S*(*_i_*)(*x*) where *x* is the closest grid point, and add Gaussian noise *Z*, corresponding to non-spatial variation between households with variance equal to the respective MCML estimate.
4. Compute the kriging predictor for *S*(*_i_*), denoted by
5. Repeat i) and ii) for *I* = 1, . . . , 10^4^ and finally approximate (2) and (3) as

Where *I*(*a* > l) is an indicator function that takes value 1 if a > *l* and 0 otherwise.

**References**

CHRISTENSEN, O. F. (2004). Monte Carlo maximum likelihood in model-based geostatistics. *Journal of Computational and Graphical Statistics*. **3**, 702-718.

CHRISTENSEN, O. F., ROBERTS, G. O. & SKÖLD, M. (2006). Robust Markov chain Monte Carlo methods for spatial generalized linear mixed models. *Journal of Computational and Graphical Statistics.* **15**, 1-17.

FANSHAWE, T. & DIGGLE, P. J. (2013). Adaptive sampling design for spatio-temporal prediction. In *Spatio-temporal designs*, J. Mateu & W. G. Müller, eds. John Wiley and Sons, Ltd, pp. 249-268.

GEYER, C. J. (1994). On the convergence of Monte Carlo maximum likelihood calculations. *Journal of the Royal Statistical Society, Series B* **56**, 261-274.

GEYER, C. J. (1996). Estimation and optimization of functions. In *Markov Chain Monte Carlo in Practice*, W. Gilks, S. Richardson & D. Spiegelhalter, eds. London: Chapman and Hall, pp. 241-258.

GEYER, C. J. (1999). Likelihood inference for spatial point processes. In *Stochastic Geometry, Likelihood and Computations.* O. E. Barndorff-Nielsen, W. S. Kendall & M. N. M. van Lieshout, eds. Boca Raton, FL; Chapman and Hall/CRC, pp. 79-140.

GEYER, C. J. & THOMPSON, E. A. (1992). Constrained Monte Carlo maximum likelihood for dependent data. *Journal of the Royal Statistical Society, Series B* **54**, 657-699.

GIORGI, E., SESAY, S. S., TERLOUW, D. J. & DIGGLE, P. J. (2015). Combining data from multiple spatially referenced prevalence surveys using generalized linear geostatistical models. *Journal of the Royal Statistical Society, Series A*. In press.

**Supplementary File 2 – Model Based Geostatistics Model Validation**

**Model Validation**

*Model validation methodology*

Model validation is directed at ascertaining whether the fitted model adequately reproduces the spatial correlation structure of the data. To achieve this, we first fitted a simple logistic regression model to the data, i.e. adjusting for the regression effects of the environmental variables but ignoring any spatial correlation. We then calculated the empirical semi-variogram of the residuals from this model, to provide an estimate of the underlying spatial correlation structure of the data. Next, we repeated the logistic regression fitting and variogram calculation from each of 10,000 datasets simulated under the fitted model. From the simulated variograms, we calculated pointwise 95% tolerance bounds for the semi-variogram under the assumption that the model generated the data. Semi-variograms that fell within the tolerance bounds were considered indicative of adequate fit to the data. Secondly, cross-validation was conducted by fitting the model to a random sample of 70% of the dataset, calculating the root-mean-square prediction error (RMSE) of prevalence over the 70% sample, and comparing this with the RMSE achieved when the fitted model was used to predict prevalence at the locations of the remaining 30% of the data.

*MBG model validation results*

Two methods to validate the model were used. The results of the probabilistic model validation for both the PCR (figure S2a) and seroprevalence (figure S2b) outcomes suggested good model fit, as in each case the empirical semi-variogram lay well within the 95% tolerance limits throughout its range. The semi-variograms also suggest that there is residual spatial dependence in both PCR and seroprevalence up to 1.5km. Secondly, the results of the cross-validation also suggested well-fitting models. For the PCR model, the MSEs of the fitting and validation sub-sets dataset were both 0.26 whereas the MSEs for the seroprevalence model were also similar (0.28 and 0.24) for the fitting and validation sub-set, respectively.

**Figure Legend**

**Figure S2:** **Probabilistic geostatistical model validation**. Semi-variogram (solid line) and predicted 95% tolerance bounds (dashed lines) for probabilistic model validation for a) PCR prevalence and b) Seropositivity


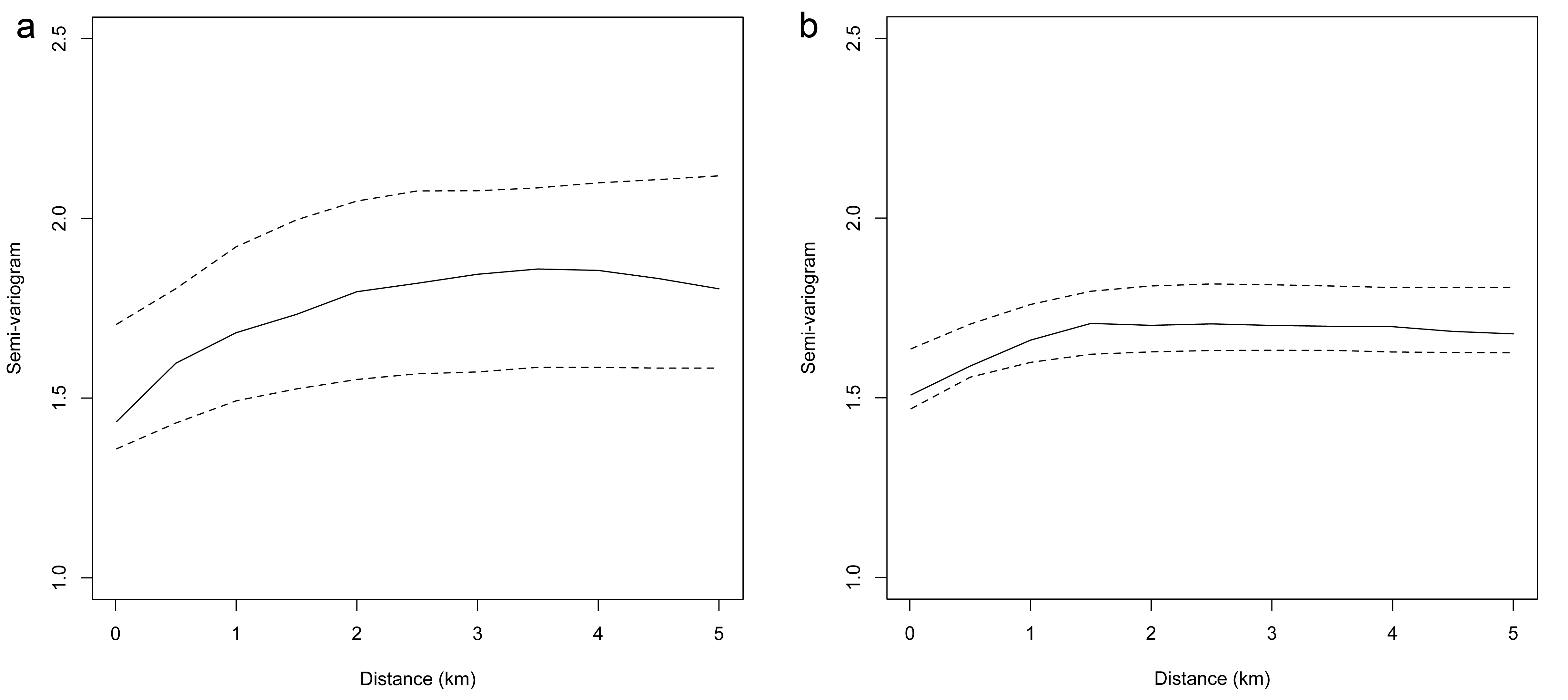


**Supplementary File – Video Legends**

**Supplementary Movie 1: Impact of sample size on hotspot boundaries: PCR.**

Animation depicting the impact of sample size on hotspot boundaries, defined using PCR positivity. The series of maps corresponds to the random subset of the actual data that was randomly selected at 10% intervals. The full sample size in the animation (100%) corresponds the 30% of the population that were sampled as part of the survey.

**Supplementary Movie 2: Impact of sample size on hotspot boundaries: Seropositivity.** Animation depicting the impact of sample size on hotspot boundaries, defined using seropositivity. The series of maps corresponds to the random subset of the actual data that was randomly selected at 10% intervals. The full sample size in the animation (100%) corresponds the 30% of the population that were sampled as part of the survey.
